# Survey of Bacterial Isolates and Their Antimicrobial Susceptibility Patterns from Dogs with Infective Endocarditis

**DOI:** 10.3390/pathogens12081011

**Published:** 2023-08-03

**Authors:** Alexander Schreiber, Steven E. Epstein, Barbara A. Byrne, Krystle L. Reagan

**Affiliations:** 1 VCA Animal Specialty Emergency Center, 1535 S Sepulveda Blvd, Los Angeles, CA 90025, USA; alexschr1313@gmail.com; 2 Department of Surgical and Radiological Sciences, School of Veterinary Medicine, University of California-Davis, Davis, CA 95616, USA; seepstein@ucdavis.edu; 3 Department of Pathology, Microbiology, and Immunology, School of Veterinary Medicine, University of California-Davis, Davis, CA 95616, USA; bbyrne@ucdavis.edu; 4 Department of Medicine and Epidemiology, School of Veterinary Medicine, University of California-Davis, Davis, CA 95615, USA

**Keywords:** sepsis, cardiac infection, endomyocarditis, bacteremia

## Abstract

Infective endocarditis (IE) is a potentially fatal disease in dogs. Limited information exists regarding the characterization of bacterial isolates from dogs with IE. The objective of this study was to describe bacterial isolates associated with IE and their antimicrobial susceptibility patterns. A retrospective analysis of dogs with IE and bacterial isolates was performed, and antimicrobial susceptibility was interpreted using current veterinary cut points where available. The susceptibility rate was assessed for association with survival and previous antimicrobial administration. Fifty-one bacterial isolates were identified from 45 dogs, and 33 had antimicrobial susceptibility performed. *Staphylococcus* spp. (14/51; 27.5%) was the most common organism. Antimicrobials with the lowest susceptibility rate were ampicillin (19/26; 73%), doxycycline (16/22; 73%), and enrofloxacin (22/29; 76%) with 12/33 (36%) of isolates exhibiting multidrug resistance (MDR). Individual antimicrobial resistances and the MDR rate were not associated with a difference in survival rate. Bacterial isolates from dogs that had received fluoroquinolone antimicrobials in the month before diagnosis had a higher rate of non-intrinsic fluoroquinolones resistance (5/8;62.5%) compared to those that did not receive fluoroquinolones (2/21; 9.5%) (*p* = 0.03). Antimicrobial resistance and MDR phenotype were common in this study. Culture and antimicrobial susceptibility testing should be pursued in dogs with IE to help guide antimicrobial therapy.

## 1. Introduction

Infective endocarditis (IE) is an uncommon but potentially fatal infection of the heart valves or endocardium, with an incidence of <1% in dogs and a mortality rate of approximately 50% [1,2,3,4]. Infective endocarditis in dogs primarily targets the aortic, mitral, and, less commonly, the tricuspid and pulmonic valves, and can result in complications including left sided congestive heart failure, arrhythmias, thromboembolic disease, glomerulonephritis, polyarthritis, and acute kidney injury [3,4,5]. A definitive diagnosis requires identification of the vegetative or erosive endocardial lesions with a biopsy [6]. However, an antemortem presumptive diagnosis is obtained by fulfilling the modified Duke criteria, which incorporates criteria for diagnosis, such as echocardiographic findings of vegetative lesions or growth from microbiological blood cultures [5,6].

The most commonly reported bacterial etiologies associated with IE in dogs include *Staphylococcus* spp., *Streptococcus* spp., *Escherichia coli*, and *Bartonella* spp. [1,2,3,4,5,7]. No consensus empiric antimicrobial therapy has been described for dogs with IE. However, broad spectrum antimicrobial treatment that targets the most commonly identified organisms is recommended. Empiric therapy commonly includes a β-lactam in conjunction with an aminoglycoside or fluoroquinolone antimicrobial [5,7,8]. Identifying the etiologic agent and corresponding antimicrobial susceptibility pattern is important in formulating and refining an appropriate antimicrobial treatment plan. However, microbiological blood cultures are negative in 24–60% of dogs with IE and there are some clinical situations where blood cultures may not be performed, making culture-directed antimicrobial therapy impossible in these instances [1,3,4,7,9,10].

## 2. Materials and Methods

### 2.1. Data Collection

Medical records from the UC Davis William R Pritchard Veterinary Medical Teaching Hospital from 2005 to 2020 were reviewed for dogs with a diagnosis of IE. Dogs were included if they had a diagnosis of IE upon necropsy evaluation with histopathology of the valve or through an anti-mortem fulfillment of the modified Duke criteria by meeting 2 major criteria, or 1 major and 2 minor criteria (Table 1) [8]. Additionally, a positive microbiological blood culture or culture of the heart valve from a specimen obtained at necropsy had to be documented to be included. The institutional protocol for the collection of blood culture specimens is to aseptically collect 3 to 6 aliquots of blood. Specimens were collected from three different anatomic locations at 0-, 10-, and 60-min time points. The specimens were then aseptically transferred to aerobic or anaerobic blood culture vials, incubated at 35 °C, and subcultured on blood agar after 24 h, 48 h, and 5 days of incubation. Isolated bacterial colonies were identified with matrix-assisted laser desorption ionization time-of-flight spectrometry analysis, standard biochemical testing including spot tests, coagulase, tubed media, and bacterial identification strips (API, Biomerieux, Marcy-l’Etoile, France), or a combination of these methodologies. The microbiological culture results were considered positive after independent review by 2 authors (K.L.R. and S.E.E.) and considered unlikely to represent contamination by both reviewers based on the presence of the same organism in ≥2 culture specimens. If only 1 specimen was documented to have bacterial growth, the cultures were considered positive if there was evidence of vegetative valvular lesions on echocardiogram and the bacterial isolate was a typical IE pathogen. Antimicrobial susceptibility was determined using standard methodology using a broth microdilution (Sensititre, ThermoFisher, Waltham, MA, USA). The minimum inhibitory concentration (MIC) was recorded for each bacteria-antimicrobial pair. Where possible, the bacterial isolates were categorized as resistant, intermediate, or susceptible based on current veterinary Clinical Laboratories Standards Institute guidelines [11,12] or extrapolated from human guidelines [13]. In instances where the isolate was not tested against antimicrobial concentrations that include current breakpoints, this was noted and susceptibility from the time of culture was utilized for further analysis in the study. Further, all isolate-antimicrobial pairs that fell into the intermediate classification were considered non-susceptible. Bacterial isolates were categorized as multi drug resistant (MDR) when resistance to one antimicrobial in ≥3 antimicrobial classes was documented where intrinsic resistance would not be expected [14]. Cases were defined as polymicrobial if >1 bacterial isolate was identified from cultures.

Data from the electronic medical record were collected for each enrolled dog, including type of culture submitted (aerobic alone or aerobic and anaerobic), specimen collected (blood or cardiac valve collected during necropsy examination), number of specimens collected, and number of those specimens that were positive for bacterial growth. Further, the signalment, antimicrobial therapy in the 1 month before IE diagnosis, empiric antimicrobial therapy after IE diagnosis but before culture results were available, and outcome were recorded. Dogs were considered non-survivors if their death or euthanasia was attributed to the IE diagnosis. Dogs were categorized as survivors if no death or euthanasia was attributed to the IE diagnosis, and they were alive at ≥1 month after IE diagnosis. Dogs that did not have follow up beyond 1 month after IE diagnosis were categorized as unknown outcome.

### 2.2. Statistical Analysis

Descriptive statistics were utilized to summarize the population demographics and determine the proportion of isolates that were susceptible to antimicrobials. Continuous variables were tested for normality with a D’Agostino-Pearson normality test and normally distributed parameters were reported with mean and standard deviation. The proportion of MDR organisms was compared between survivors and non-survivors using a Fisher’s exact test. The rate of antimicrobial resistance between dogs that had and had not been exposed to an antimicrobial class in the 1 month before IE diagnosis was compared if ≥3 bacterial isolates were available from dogs that had received an antimicrobial in that class using a Fisher’s exact test. A Bonferroni-Dunn correction was applied to multiple comparisons when statistical significance was observed after the initial comparison and the adjusted *p* value was reported. A commercially available statistical software was used for analysis (GraphPad version 9.5.0, Prism, San Diego, CA, USA). For all comparisons, *p* < 0.05 was considered significant.

## 3. Results

### 3.1. Population Description

During the study period, 113 dogs were diagnosed with IE based on necropsy findings or antemortem fulfillment of the modified Duke criteria. Of these dogs, 45/113 (40%) had a bacterial etiologic agent isolated and were included in the study. The mean age of the included dogs was 8.3 years (standard deviation (SD): 3.4 years). The sex distribution included 27 (60%) neutered males, 3 (6.5%) intact males, 13 (31%) neutered females, and 2 (4.35%) intact females. Dog breeds with ≥2 dogs represented included 12 (26%) Labrador retrievers or Labrador retriever hybrids, 4 (8.7%) golden retrievers, 4 (8.7%) pit bull or pit bull hybrids, 3 (6.5%) German shepherd dogs, 2 (4.4%) Maltese, and 2 (4.4%) Rhodesian ridgebacks. Thirty (66.6%) of the study dogs were characterized as non-survivors, 13 (28.8%) as survivors, and 2 (4.4%) had an unknown outcome.

### 3.2. Microbiological Cultures and Bacterial Isolates

Of the 45 dogs with positive microbiological cultures, bacterial isolates were obtained from antemortem blood cultures in 41/45 (91%) dogs and from cultures of the affected valve collected at necropsy from 4/45 (8.9%) dogs. Antemortem blood cultures included 33 dogs with aerobic and anaerobic blood cultures, and 8 dogs with only aerobic blood cultures performed. From these 45 dogs, 51 bacterial isolates were identified (Table 2). Thirty gram-positive aerobic organisms were identified from 29 dogs and 13 gram-negative aerobic isolates were obtained from 11 dogs. Eight anaerobic bacterial isolates were obtained from 8 dogs (Table 2).

Forty-one dogs were diagnosed with a monomicrobial infection and 4 with polymicrobial infections. Three of the 4 polymicrobial infections were diagnosed based on antemortem blood cultures, and 1 was obtained from a culture of the valve collected at necropsy. The four polymicrobial infections included 1 dog with *Escherichia coli* and *Enterococcus faecium*; 1 dog with *Clostridium perfringens* and *Streptococcus viridans*; 1 dog with *Staphylococcus pseudintermedius, Enterococcus faecalis*, and *E. coli*; and 1 dog with *Pseudomonas aeruginosa, Enterobacter cloacae*, and *E. coli*.

In non-survivors, the most common bacterial isolates were *Streptococcus* spp. (9/30, 30%) and *Staphylococcus* spp. (8/30, 27%). In survivors, the most common bacterial isolates were *Staphylococcus* spp. (5/10, 50%) and *Enterococcus* spp. (3/10, 30%). There was no difference in the proportion of dogs with gram-positive, gram-negative, anaerobic isolates, or mixed infections between survivors and non-survivors (*p* = 0.67).

### 3.3. Antimicrobial Susceptibility Patterns

Of the 51 bacterial isolates, 33 bacterial isolates from 31 dogs had associated antimicrobial susceptibility performed. Eighteen bacterial isolates from 14 dogs did not have antimicrobial susceptibility available. These included 7 isolates that were presumed to have broad susceptibility based on the species identification (5 *Streptococcus* spp. isolates and 2 anaerobic isolates), 4 isolates that were obtained from necropsy specimens where antimicrobial susceptibility is not routinely performed in our institution, and 7 isolates that did not have antimicrobial susceptibility performed for unknown reasons.

The rate of antimicrobial susceptibility is outlined in Table 3 with MIC data outlined for bacterial species with >1 isolate in Table S1. Of the 13 *Staphylococcus* spp. isolates with available culture and susceptibility results, the antimicrobial with the highest rate of non-intrinsic resistance was ampicillin (7/13, 54%). Four of the 13 (31%) *Staphylococcus* spp. isolates were oxacillin resistant. Isolates from survivors and non-survivors did not significantly differ in the rate of resistance to individual antimicrobials. Multidrug resistance was noted in 12/33 (36%) isolates. This consisted of 5 *Staphylococcus* spp., 4 *Enterococcus* spp., 2 *Streptococcus* spp., and 1 *E. coli*. There was no difference in the rate of MDR isolates between survivors and non-survivors (*p* > 0.99).

Thirty of the 45 dogs had been administered antimicrobial therapy within the 1 month before IE diagnosis. Administration of antimicrobials within 1 month prior to diagnosis was not associated with survivor status (*p* = 0.7). Antimicrobial susceptibility testing was available for 23 isolates from 21 dogs that had received previous antimicrobials. The rate of isolation of at least one MDR organism did not differ between those that had received prior antimicrobials (8/21; 38%) compared to those without previous antimicrobial administration (3/10; 30%) (*p* > 0.99).

Dogs that had received a fluoroquinolone antimicrobial in the 1 month before the diagnosis of IE had a higher rate of isolates with non-intrinsic resistance to that antimicrobial class (5/8; 72%) when compared to dogs that had not received a fluoroquinolone antimicrobial in the 1 month before IE diagnosis (2/20, 29%) (adjusted *p* = 0.027) (Table 4). Administration of β-lactam antimicrobials and tetracyclines in the 1 month before diagnosis of IE was not associated with isolation of bacteria with resistance to the corresponding classes of antimicrobials.

### 3.4. Empiric Antimicrobial Therapy

Of the 45 dogs in the study, 43 (96%) were treated with antimicrobials before culture results were available. Of the 43 cases, the most prescribed empiric antimicrobials included a penicillin with or without a β-lactamase inhibitor (40/43, 93%), a fluoroquinolone (33/43, 77%), or a combination including antimicrobials from these classes 31/43 (72%) (Table S2). Of the 31 dogs that had bacterial isolates with antimicrobial susceptibility performed, 3/31 (9.7%) dogs had bacterial isolates that were resistant to all the antimicrobials used in the empiric antimicrobial therapy. This included 1 dog treated empirically with ampicillin-sulbactam alone, 1 with ampicillin and enrofloxacin, and 1 with ampicillin-sulbactam and enrofloxacin. Two of these dogs were non-survivors and one was a survivor.

When assessing commonly prescribed empiric IE therapy and susceptibility, 1/31 dogs with antimicrobial susceptibility had a bacterial isolate, a *Pseudomonas aeruginosa*, that had predicted resistance to both drugs in the combination ampicillin-sulbactam and enrofloxacin. No dogs had isolates with resistance to both of the antimicrobials in the combination ampicillin and amikacin.

## 4. Discussion

Here we describe the bacterial isolates associated with IE in dogs and the corresponding antimicrobial susceptibility patterns. Slightly more than one-third of the organisms isolated in this study exhibited an MDR phenotype. Three dogs in this study were treated with empiric antimicrobials that were determined to be inadequate based on subsequent culture and antimicrobial susceptibility results. However, there was no difference in the rate of MDR isolates or resistance to individual antimicrobials between those that did and did not survive IE. Further, we observed an increased rate of isolates with non-intrinsic resistance to fluoroquinolones in dogs that had received an antimicrobial in that class in the 1 month before diagnosis of IE. These findings highlight the utility of culture and antimicrobial susceptibility testing in directing antimicrobial therapy for dogs with IE.

The most commonly isolated organisms in this study were *Staphylococcus* spp. and *Streptococcus* spp. This is similar to recent veterinary studies where *Staphylococcus* spp. was isolated in 15–25% of cases and *Streptococcus* spp. in 10–29% of cases [3,4,5,7,15]. *Escherichia coli* was the third most common isolate (16%) in our study, which has been noted in other studies [1,15]. These organisms are similar to findings in humans where *Staphylococcus aureus*, *Streptococcus* spp., and *Enterococcus* spp. are the most commonly identified bacterial pathogens associated with IE [16,17,18]. *Bartonella*, a gram-negative, aerobic fastidious organism, is also implicated as a cause of IE and often tested as part of the workup for IE, especially in cases with negative blood cultures [1,3,4]. Our study did not assess the rate of *Bartonella* IE, as the culture methodology is different and this organism is not isolated with routine blood cultures.

No single antimicrobial had a susceptibility rate that was >90%. This finding is similar to other surveys of bacterial infections in dogs, including urinary tract infections and pyoderma, in which resistance to commonly administered antimicrobials, including potentiated β-lactams and fluoroquinolones, are frequently documented [19,20,21,22]. Of note, the presence of antimicrobial resistance was not correlated with the disease outcome, meaning that the likelihood of a dog dying from complications of IE, such as heart failure, thromboembolic disease, or euthanasia due to the poor prognosis, is independent of antimicrobial resistance status.

An MDR phenotype was noted in 36% of the isolates in our study. To the authors’ knowledge, no previous assessment of the MDR rate of bacteria from dogs with IE has been published for comparison; however, a similar rate of MDR phenotypes has been observed in bacterial isolates causing bacteremia, urinary tract infections, pneumonia, and pyoderma [19,20,21,22,23,24,25]. The high rate of MDR phenotype may reflect the patient population that is cared for at our institution, a tertiary referral hospital, which may have an increased rate of chronic illness or previous antimicrobial exposure. In people with MDR IE, there are associated increases in mortality, length of hospital stay, and cost of care [26]. No difference in the rate of individual antimicrobial susceptibility or MDR phenotype status was noted between dogs that did or did not survive IE. However, with the limited sample size, this study was likely not powered to robustly detect any potential differences, so this must be cautiously interpreted. Large scale, multi-institution studies are warranted to further investigate these findings.

While no consensus guidelines are published indicating the most effective empiric antimicrobial therapy for dogs with IE, guidelines for people with IE recommend β-lactam antimicrobials in combination with an aminoglycoside or fluoroquinolone as first line therapy [16]. The combination of a fluoroquinolone and a β-lactam antimicrobial are the most commonly prescribed empiric antimicrobials in dogs with IE [7,27]. Of the 33 isolates in our study, 76% were susceptible to enrofloxacin and 73% susceptible to ampicillin. One of the 31 dogs in our study with available antimicrobial susceptibility had isolates that were resistant to both antimicrobials used in this combination, but no dogs had isolates that were resistant to both an aminoglycoside and ampicillin. Interestingly, in people with IE caused by a gram-negative organism, poorer outcomes were recorded for patients treated with combination antimicrobial therapy that included a fluoroquinolone, but the cause of this finding is not known [28]. Our study did not have an adequate sample size to compare outcomes based on treatment. Further studies may be warranted to determine the ideal empiric antimicrobial treatment options for IE in dogs, but our results support the continued utilization of β-lactam antimicrobials in combination with an aminoglycoside or fluoroquinolone as first line therapy.

Antimicrobial administration in the month before IE diagnosis was common. Studies in people have demonstrated that antimicrobial administration can change resistance patterns in the microbiota for up to 3 months post treatment [29]. Further, previous administration of antimicrobials may be associated with an increased rate of subsequent antimicrobial resistant infections [30,31,32,33]. In our study, there was no significant association with previous antimicrobial administration and the rate of MDR phenotype isolates. However, an increased rate of non-intrinsic resistance to fluroquinolone antimicrobials was noted in dogs that had been administered a fluoroquinolone in the 1 month before diagnosis of IE. This finding has been noted in human and veterinary studies where prior exposure has been associated with the isolation of fluoroquinolone resistant bacteria [34,35,36,37,38]. The increased rate of isolation of fluoroquinolone-resistant organisms in our study may be due to selection bias, as dogs that had received a fluoroquinolone in the 1 month before IE diagnosis may have had false-negative blood cultures if the causative organism was fluoroquinolone susceptible, therefore biasing outcomes. However, this finding was not observed for other antimicrobial classes. An alternative explanation is the acquisition of fluoroquinolone resistance due to selection pressure. Fluoroquinolone resistance occurs through a variety of mechanisms but mainly due to target-site mutations in topoisomerases and quinolone resistance-determining regions resulting in mutations within gyrA and parC genes which reduce binding efficiency of fluoroquinolones [39,40,41]. Other reported mechanisms implicated in resistance include changes to membrane permeability and expression of efflux pumps [39]. A thorough history should be obtained for any previous antimicrobial exposure, as that exposure may be associated with a subsequent increased rate of resistance, and further highlights the need for culture directed antimicrobial therapy.

There were several limitations to this study. Culture and susceptibility data were not available for 14 isolates, which may have impacted the rate of antimicrobial resistance reported here. Importantly, some organisms did not have susceptibility performed as they were presumed to have broad susceptibility, such as *Streptococcus* spp., which likely introduced bias into the analysis. Also, 60% of dogs considered for enrollment into this study had IE but etiologic and antimicrobial susceptibility data were lacking and the cases were excluded from analysis. Some dogs may have had negative culture results due to prior antimicrobial usage resulting in false negatives, possibly skewing our results to more resistant isolates. Furthermore, it is hard to determine a cause-and-effect relationship in regard to the bacterial isolates from cases with polymicrobial results. Some of the identified organisms may be contaminants or not contributing to the pathogenesis of IE and this may skew the isolate results. Additionally, dogs with IE caused by fastidious organisms, such as *Bartonella* spp., were not included in this study as they did not meet the enrollment criteria of a positive culture. Also, blood culture and antimicrobial susceptibility testing methodology changed over the timespan of this study, including antimicrobial break points. To address this, isolate susceptibility results were reinterpreted using current standards where possible. In some cases, antimicrobial dilutions did not encompass the currently accepted breakpoints and susceptibility was interpreted from breakpoints available at the time of initial culture. Further, the sample size was limited in this study, making statistical comparisons between groups difficult. Larger scale, multi-institution studies may result in more robust datasets that allow for comparisons between groups more readily and allow for prospective assessment of resistance patterns and how they change over time.

## 5. Conclusions

This study demonstrated an MDR phenotype in over one-third of the isolates obtained from dogs with IE. Of all the isolates with available culture and sensitivity results, no single antimicrobial tested had >90% susceptibility rate to all isolates; however, the rate of individual antimicrobial resistance did not appear to be strongly correlated with the disease outcome. There may be an effect of previously prescribed antimicrobials on antimicrobial susceptibility; therefore, a thorough history of previously prescribed antimicrobials should be considered when making an empiric antimicrobial plan. The commonly used empiric antimicrobial therapy of β-lactams and fluoroquinolones or β-lactam and aminoglycosides would have provided adequate antimicrobial coverage for most isolates in this study. Microbiological blood culture should be pursued in cases of suspected IE to help refine antimicrobial therapy. Future studies investigating pathogen-specific antimicrobial recommendations should be conducted, as is seen in human medicine [16].

## Figures and Tables

**Table 1:** Modified Duke criteria describing major and minor criteria for the antemortem diagnosis of IE in dogs [8].

Major Criteria	Minor Criteria
Echocardiogram lesions (vegetative, erosive, abscess, more than trivial valvular insufficiency) consistent with IE	Rectal temperature > 39 °C
	New or worsening heart murmur
Positive blood cultures with	Predisposing cardiac disease (subaortic stenosis)
≥2 bottles with typical organism	Evidence of thromboembolic disease
≥3 bottles with common skin contaminant	Evidence of secondary immune-mediated disease
Persistent positive cultures ≥ 12 h	Microbiological findings not meeting major criteria
	Positive *Bartonella* spp. serology

**Table 2:** Total bacterial species isolated and isolates subcategorized by survivor status of the dog with IE.

	All Isolates %(n)	Survivor Subcategory %(n)	Non-Survivors Subcategory %(n)	Lost to Follow Up Subcategory %(n)
**Total isolates**	51	15	34	2
**Gram positive aerobes**				
*Staphylococcus* spp.	27.5% (14)	33.3% (5)	23.5% (8)	50% (1)
*Streptococcus* spp.	21.6% (11)	13.3% (2)	26.5% (9)	-
*Enterococcus* spp.	9.8% (5)	20.0% (3)	5.9% (2)	-
**Gram negative aerobes**				
Enteric				
*Escherichia coli*	15.7% (8)	13.3% (2)	17.6% (6)	-
*Enterobacter cloacae*	2.0% (1)	-	3.0% (1)	-
*Salmonella* sp.	2.0% (1)	6.7% (1)	-	-
*Serratia marcescens*	2.0% (1)	-	-	50% (1)
Non-enteric				
*Pseudomonas* spp.	3.9% (2)	-	5.9% (2)	-
**Facultative Anaerobes**				
*Pasteurella* spp.	5.9% (3)	-	8.9% (3)	-
**Anaerobes**				
*Actinomyces* sp.	5.9% (3)	6.7% (1)	5.9% (2)	-
*Clostridium perfringens*	2.0% (1)	-	3.0% (1)	-
*Leptotrichia* sp.	2.0% (1)	6.7% (1)	-	-

**Table 3:** Antimicrobial susceptibility rate for various antimicrobial agents and corresponding survivor status for the dog from which the isolate was obtained.

Antimicrobial	Isolates Tested n	All Organisms n (%)	Survivor n (%)	Non-Survivor n (%)	*p* Value
Amikacin	29	23 (79%)	7 (64%)	11 (69%)	0.3
Amoxicillin/clavulanic acid	26	23 (88%)	11 (100%)	10 (77%)	0.2
Ampicillin	26	19 (73%)	7 (64%)	10 (77%)	0.7
Cefazolin	27	21 (78%)	9 (90%)	11 (73%)	0.6
Chloramphenicol	32	29 (90%)	12 (86%)	15 (94%)	0.6
Clindamycin	19	16 (84%)	5 (71%)	10 (91%)	0.5
Doxycycline	22	16 (73%)	7 (58%)	8 (89%)	0.2
Enrofloxacin	29	22 (76%)	8 (73%)	12 (75%)	1
Imipenem	30	27 (90%)	12 (92%)	13 (87%)	1
Marbofloxacin	26	23 (88%)	9 (90%)	12 (86%)	1
TMS	28	24 (86%)	10 (91%)	12 (80%)	0.6
Vancomycin	9	9 (100%)	5 (100%)	4 (100%)	1

**Table 4:** Isolate susceptibility stratified on exposure to an antimicrobial in that class in the 1 month before IE diagnosis.

Antimicrobial Class	No. Dogs Administered Specified Antimicrobial	% (n) of Dogs Administered Specified Antimicrobial with ≥1 Isolate Exhibiting Non-Intrinsic Resistance to Antimicrobial	No. Dogs Not Administered Specified Antimicrobial	% (n) of Dogs Not Administered Specified Antimicrobial with ≥1 Isolate Exhibiting Non-Intrinsic Resistance to Antimicrobial	Adjusted *p* Value
β-lactams	11	27% (3)	20	67% (6)	1
Fluoroquinolones	8	72% (5)	20	29% (2)	0.027
Tetracyclines	4	25% (1)	26	89% (8)	0.54

## Data Availability

Further inquiries can be directed to the corresponding authors.

## Supplementary Materials

The following supporting information can be downloaded at: https://www.mdpi.com/article/10.3390/pathogens12081011/s1, Table S1: Antimicrobial susceptibility data with corresponding minimum inhibitory concentrations for bacterial species with more than 4 isolates; Table S2: Empiric antimicrobial therapy administered to dog with infective endocarditis.

## References

[1] MacDonald K.A., Chomel B.B., Kittleson M.D., Kasten R.W., Thomas W.P., Pesavento P. (2004). A prospective study of canine infective endocarditis in northern California (1999–2001): Emergence of Bartonella as a prevalent etiologic agent. JVIM.

[2] Calvert C.A., Greene C.E., Hardie E.M. (1985). Cardiovascular infections in dogs: Epizootiology, clinical manifestations, and prognosis. JAVMA.

[3] Reagan K.L., Visser L.C., Epstein S.E., Stern J.A., Johnson L.R. (2022). Outcome and prognostic factors in infective endocarditis in dogs: 113 cases (2005–2020). JVIM.

[4] Sykes J.E., Kittleson M.D., Chomel B.B., MacDonald K.A., Pesavento P.A. (2006). Clinicopathologic findings and outcome in dogs with infective endocarditis: 71 cases (1992–2005). JAVMA.

[5] MacDonald K. (2010). Infective endocarditis in dogs: Diagnosis and therapy. Vet. Clin. N. Am. Small Anim. Pract..

[6] MacDonald K.A., Bonagura J.D., Twedt D.C. (2014). Infective endocarditis. Kirk’s Current Veterinary Therapy XV (Small Animal Practice).

[7] Berrezaie M., Connolly D., Cruzado J., Mederska E., Dukes-McEwan J., Humm K. (2023). Infective endocarditis in dogs in the UK: 77 cases (2009–2019). J. Small Anim. Pract..

[8] Ljungvall I., Häggström J. (2017). Adult-onset valvular heart disease. Textbook of Veterinary Internal Medicine Expert Consult.

[9] Sisson D., Thomas W.P. (1984). Endocarditis of the aortic valve in the dog. JAVMA.

[10] Kilkenny E., Watson C., Dukes-McEwan J., Bode E.F., Hezzell M.J., Payne J.R., Borgeat K. (2021). Evaluation of serum cardiac troponin-I concentrations for diagnosis of infective endocarditis in dogs. JVIM.

[11] (2020). CLSIPerformance Standards for Antimicrobial Disk Dilution Susceptibility Tests for Bacteria Isolated from Animals 5th ed CLSI Supplement.

[12] (2017). CLSI Methods for Antimicrobial Susceptibility Testing of Infrequently Isolated or Fastidious Bacteria Isolated from Animals, 1st ed..

[13] CLSI (2020). Performance Standards for Antimicrobial Susceptibility Testing.

[14] Magiorakos A.P., Srinivasan A., Carey R.B., Carmeli Y., Falagas M.E., Giske C.G., Harbarth S., Hindler J.F., Kahlmeter G., Olsson-Liljequist B. (2012). Multidrug-resistant, extensively drug-resistant and pandrug-resistant bacteria: An international expert proposal for interim standard definitions for acquired resistance. Clin. Microbiol. Infect..

[15] Peddle G.D., Drobatz K.J., Harvey C.E., Adams A., Sleeper M.M. (2009). Association of periodontal disease, oral procedures, and other clinical findings with bacterial endocarditis in dogs. JAVMA.

[16] Baddour L.M., Wilson W.R., Bayer A.S., Fowler Jr V.G., Tleyjeh I.M., Rybak M.J., Barsic B., Lockhart P.B., Gewitz M.H., Levison M.E. (2015). Infective Endocarditis in Adults: Diagnosis, Antimicrobial Therapy, and Management of Complications. Circulation.

[17] Di Domenico E.G., Rimoldi S.G., Cavallo I., D’Agosto G., Trento E., Cagnoni G., De Vecchi E. (2019). Microbial biofilm correlates with an increased antibiotic tolerance and poor therapeutic outcome in infective endocarditis. BMC Microbiol..

[18] Van der Meer J.T.M., Van Vianen W., Hu E., Van Leeuwen W.B., Valkenburg H.A., Thompson J., Michel M.F. (1991). Distribution, antibiotic susceptibility and tolerance of bacterial isolates in culture-positive cases of endocarditis in The Netherlands. Eur. J. Clin. Microbiol. Infect. Dis.

[19] Huerta B., Maldonado A., Ginel P.J., Tarradas C., Gómez-Gascón L., Astorga R.J., Luque I. (2011). Risk factors associated with the antimicrobial resistance of staphylococci in canine pyoderma. Vet. Microbiol..

[20] Lai C.-H., Ma Y.-C., Shia W.-Y., Hsieh Y.-L., Wang C.-M. (2022). Risk Factors for Antimicrobial Resistance of Staphylococcus Species Isolated from Dogs with Superficial Pyoderma and Their Owners. Vet. Sci..

[21] Wong C., Epstein S.E., Westropp J.L. (2015). Antimicrobial susceptibility patterns in urinary tract infections in dogs (2010–2013). JVIM.

[22] Yu Z., Wang Y., Chen Y., Huang M., Wang Y., Shen Z., Li G. (2020). Antimicrobial resistance of bacterial pathogens isolated from canine urinary tract infections. Vet. Microbiol..

[23] Qekwana D.N., Naidoo V., Oguttu J.W., Odoi A. (2020). Occurrence and Predictors of Bacterial Respiratory Tract Infections and Antimicrobial Resistance Among Isolates From Dogs Presented With Lower Respiratory Tract Infections at a Referral Veterinary Hospital in South Africa. Front. Vet. Sci..

[24] Saarenkari H.K., Sharp C.R., Smart L. (2022). Retrospective evaluation of the utility of blood cultures in dogs (2009–2018): 45 cases. J. Vet. Emerg. Crit. Care.

[25] Mavrides D.E., Morgan A.L., Na J.G., Graham P.A., McHugh T.D. (2022). Antimicrobial resistance profiles of bacteria associated with lower respiratory tract infections in cats and dogs in England. Vet. Rec..

[26] Falcone M., Tiseo G., Durante-Mangoni E., Ravasio V., Barbaro F., Ursi M.P., Venditti M. (2018). Risk factors and outcomes of endocarditis due to non-HACEK gram-negative bacilli: Data from the prospective multicenter Italian endocarditis study cohort. Antimicrob. Agents. Chemother..

[27] De Briyne N., Atkinson J., Borriello S.P., Pokludová L. (2014). Antibiotics used most commonly to treat animals in Europe. Vet. Rec..

[28] Veve M.P., McCurry E.D., Cooksey G.E., Shorman M.A. (2020). Epidemiology and outcomes of non-HACEK infective endocarditis in the southeast United States. PLoS ONE.

[29] Bakhit M., Hoffmann T., Scott A.M., Beller E., Rathbone J., Del Mar C. (2018). Resistance Decay in Individuals after Antibiotic Exposure in Primary Care: A Systematic Review and Meta-Analysis. BMC Med..

[30] Hillier S., Roberts Z., Dunstan F., Butler C., Howard A., Palmer S. (2007). Prior antibiotics and risk of antibiotic-resistant community-acquired urinary tract infection: A case–control study. J. Antimicrob. Chemother..

[31] Paschke A.A., Zaoutis T., Conway P.H., Xie D., Keren R. (2010). Previous Antimicrobial Exposure Is Associated With Drug-Resistant Urinary Tract Infections in Children. Pediatrics.

[32] Gibson J.S., Morton J.M., Cobbold R.N., Sidjabat H.E., Filippich L.J., Trott D.J. (2008). Multidrug-resistant *E. coli* and Enterobacter extraintestinal infection in 37 dogs. JVIM.

[33] Hernandez J., Bota D., Farbos M., Bernardin F., Ragetly G., Médaille C. (2014). Risk factors for urinary tract infection with multiple drug-resistant Escherichia coli in cats. J. Feline Med. Surg..

[34] Gasink L.B., Fishman N.O., Weiner M.G., Nachamkin I., Bilker W.B., Lautenbach E. (2006). Fluoroquinolone-resistant Pseudomonas aeruginosa: Assessment of risk factors and clinical impact. Am. J. Med..

[35] Dan S., Shah A., Justo J.A., Bookstaver P.B., Kohn J., Albrecht H., Al-Hasan M.N. (2016). Prediction of Fluoroquinolone Resistance in Gram-Negative Bacteria Causing Bloodstream Infections. Antimicrob. Agents Chemother..

[36] Wedley A.L., Dawson S., Maddox T.W., Coyne K.P., Pinchbeck G.L., Clegg P., Nuttall T., Kirchner M., Williams N.J. (2017). Carriage of antimicrobial resistant Escherichia coli in dogs: Prevalence, associated risk factors and molecular characteristics. Vet. Microbiol..

[37] Leite-Martins L.R., Mahú M.I., Costa A.L., Mendes A., Lopes E., Mendonça D.M., Niza-Ribeiro J.J., de Matos A.J., da Costa P.M. (2014). Prevalence of antimicrobial resistance in enteric Escherichia coli from domestic pets and assessment of associated risk markers using a generalized linear mixed model. Prev. Vet. Med..

[38] Sato T., Yokota S., Ichihashi R., Miyauchi T., Okubo T., Usui M., Fujii N., Tamura Y. (2014). Isolation of Escherichia coli strains with AcrAB-TolC efflux pump-associated intermediate interpretation or resistance to fluoroquinolone, chloramphenicol and aminopenicillin from dogs admitted to a university veterinary hospital. J. Vet. Med. Sci..

[39] Redgrave L.S., Sutton S.B., Webber M.A., Piddock L.J. (2014). Fluoroquinolone resistance: Mechanisms, impact on bacteria, and role in evolutionary success. Trends Microbiol..

[40] Hooper D.C. (2000). Mechanisms of action and resistance of older and newer fluoroquinolones. Clin. Infect. Dis..

[41] Piddock L.J. (1999). Mechanisms of fluoroquinolone resistance: An update 1994–1998. Drugs.

